# How central and peripheral vision influence focal and ambient processing during scene viewing

**DOI:** 10.1167/jov.22.12.4

**Published:** 2022-11-02

**Authors:** Jatheesh Srikantharajah, Colin Ellard

**Affiliations:** 1 Department of Psychology, University of Waterloo, Waterloo, Canada

**Keywords:** peripheral vision, central vision, fixations, focal processing, ambient processing

## Abstract

Central and peripheral vision carry out different functions during scene processing. The ambient mode of visual processing is more likely to involve peripheral visual processes, whereas the focal mode of visual processing is more likely to involve central visual processes. Although the ambient mode is responsible for navigating space and comprehending scene layout, the focal mode gathers detailed information as central vision is oriented to salient areas of the visual field. Previous work suggests that during the time course of scene viewing, there is a transition from ambient processing during the first few seconds to focal processing during later time intervals, characterized by longer fixations and shorter saccades. In this study, we identify the influence of central and peripheral vision on changes in eye movements and the transition from ambient to focal processing during the time course of scene processing. Using a gaze-contingent protocol, we restricted the visual field to central or peripheral vision while participants freely viewed scenes for 20 seconds. Results indicated that fixation durations are shorter when vision is restricted to central vision compared to normal vision. During late visual processing, fixations in peripheral vision were longer than those in central vision. We show that a transition from more ambient to more focal processing during scene viewing will occur even when vision is restricted to only central vision or peripheral vision.

## Introduction

Central and peripheral vision have long been theorized to be part of distinct yet complementary visual systems – focal and ambient vision (Trevarthen, 1968). Ambient vision navigates the space around the body and involves the visual periphery, whereas focal vision processes detailed information through orienting central vision to selected regions of the visual field. Through the periphery, ambient visual processing provides information about the layout of the surrounding environment (Alfano & Michel, 1990), along with any motion within the setting (Finlay, 1982). Focal activity builds on ambient information, with central vision engaging in processing detailed information from salient regions of space.

Functional differences between central and peripheral vision may influence their relative importance within different periods during the time course of scene processing. Peripheral vision is particularly suited to certain tasks that have higher importance during early scene processing. Rapid (approximately 100 ms) processing of scene gist is particularly effective in peripheral vision (Larson & Loschky, 2009), even when stimuli are exclusively presented in the far periphery (Boucart, Moroni, Thibaut, Szaffarczyk, & Greene, 2013). Gist processing for certain objects, such as animals, can occur extremely quickly in the periphery (Thorpe, Gegenfurtner, Fabre-Thorpe, & Bulthoff, 2001). Peripheral vision is also sufficient for tasks involved in rapid facial processing, including identifying emotions (Rigoulot, D'Hondt, Defoort-Dhellemmes, Despretz, Honoré, & Sequeira, 2011) and forming judgments related to attractiveness (Guo, Lin, & Roebuck, 2011; Kuraguchi & Ashida, 2015). Moreover, rapid judgments formed through central vision are influenced by information gained automatically through the visual periphery (Peyrin, Roux-Sibilon, Trouilloud, Khazaz, Joly, Pichat, Boucart, Krainik, & Kauffmann, 2021), likely due to a peripheral advantage in processing speed (Carrasco, McElree, Denisova, & Giordano, 2003). In contrast to early processing, many of the tasks involved during late scene processing are more suited to central vision. As people view scenes for extended periods of time, there is a transition from initial processing of gist and layout toward detailed examination of items of interest (Antes, 1974), heavily involving the fovea. Although the function of peripheral vision with regards to early visual processing is a well-trodden subject, its role during late visual processing has been less frequently investigated, particularly in the context of scene processing.

Prior work suggests that there are distinct patterns of eye movements during scene processing between early and late time intervals that correspond to ambient and focal visual processing (Pannasch, Helmert, Roth, Herbold, & Walter, 2008; Unema, Pannasch, Joos, & Velichkovsky, 2005). Ambient visual processing occurs during the initial 2 seconds of scene viewing and predominantly involves larger saccades and shorter fixations (Pannasch et al., 2008). This initial period is followed by a focal mode of processing characterized by smaller saccades and longer fixations, particularly on salient items. This pattern indicates a transition from visual exploration through scanning to identify layout and determine salient regions, to foveation of various elements of the scene. Under normal visual conditions, mean fixation durations increase during the time course of scene processing, whereas saccade amplitudes decrease (Antes, 1974; Irwin & Zelinsky, 2002). The time course of visual processing is also affected by novel events or visible changes to an environment. When new events occur, the visual system engages in ambient visual processing, conducting exploratory eye movements toward the region of visual change (Eisenberg & Zacks, 2016).

A second way to demarcate focal and ambient processing is through the classification of fixations. One approach identifies fixations on the basis of the amplitude of the preceding saccade (Pannasch & Velichkovsky, 2009). Ambient fixations are defined as fixations preceded by saccades with an amplitude greater than 5 degrees, whereas focal fixations are preceded by a saccade with an amplitude less than 5 degrees. Larger preceding saccades are theorized to indicate fixations directed at processing spatial layout, whereas smaller preceding saccades indicate fixations aimed at nearby objects and important features. This reflects divisions in the visual field, as a saccade greater than 5 degrees would travel to an area outside of the parafovea. A similar classification strategy was used in a study involving macaque monkeys, where fixations were identified as object fixations when a fixation was within 1.5 degrees of an object in a scene, whereas fixations with a greater distance to an object were categorized as background fixations (Ito, Yamane, Suzuki, Maldonado, Fujita, Tamura, & Grün, 2017). During the course of scene viewing, macaque monkey eye movements transitioned from primarily background to object fixations, indicating a change from an ambient to a focal mode of visual processing.

Because the function of eye movements during ambient visual processing is to facilitate visual exploration, whereas eye movements during focal visual processing facilitate detailed examination, changes in eye movement patterns and broader viewing strategies during the time course of visual processing might reflect changes in the relative influence of central and peripheral vision. However, although peripheral vision may be increasingly influential during early scene processing, the function of peripheral vision for visual activity during late scene processing must be further explored. Prior studies masking peripheral or central information found consistent effects on saccade amplitudes, but mixed results for fixation durations. Saccade amplitudes increase with the size of the available visual field (Loschky & McConkie, 2002; Rayner, Inhoff, Morrison, Slowiaczek, & Bertera, 1981), whereas there are reduced saccade amplitudes when visual information is restricted to central vision (Shioiri & Ikeda, 1989), and increased amplitudes when vision is limited to the periphery (Laubrock, Cajar, & Engbert, 2013; van Diepen, 2002). However, differences in saccade amplitudes due to restrictions of the visual field may be smaller in virtual reality compared to computer-based studies with 2D environments (David, Lebranchu, Da Silva, & Le Callet, 2020).

Increases in fixation durations have been found in some visual search experiments when vision is restricted to either peripheral vision or central vision compared to normal vision (Cornelissen, Bruin, & Kooijman, 2005; Nuthmann, 2014). This may be explained by an increase in task difficulty, with people fixating longer to extract the more limited visual information that is available. Other studies masking either low or high spatial frequency information in central or peripheral vision find that increases in fixation durations relative to control only occur when critical visual information is present (Cajar, Engbert, & Laubrock, 2016; Laubrock et al., 2013). In these experiments, masking of high spatial frequencies in the periphery or low spatial frequencies in central vision had little impact on fixation durations, whereas masking of low spatial frequencies in the periphery or high spatial frequencies in central vision were associated with longer fixations when compared to normal vision. However, there are other experiments where fixation durations have decreased when vision is restricted to peripheral vision (David et al., 2019; Henderson et al., 1997) or central vision, although there may be an increase in fixation durations as more information in the periphery is occluded (David et al., 2019). There are also mixed results when contrasting fixation durations between central and peripheral vision, with some studies finding longer fixations for central vision (David et al., 2019), some finding no differences (Laubrock et al., 2013), and others finding longer fixations for peripheral vision (Cajar et al., 2016; van Diepen & d'Ydewalle, 2003). Thus, the relationship between central and peripheral vision and fixation durations requires further examination.

With regard to the relationship between central and peripheral vision and changes in eye movement patterns during the time course of scene processing, one study investigated visual search patterns in virtual reality (VR), contrasting eye movements between a scanning phase and verification phase. The length of these phases lasted variable lengths of time, depending on how long it took to locate and then refixated on a target (David, Beitner, & Võ, 2020). For any trial, the scanning or verification phase could comprise a period of time ranging from less than 2.5 seconds to greater than 7 seconds. Surprisingly, this study did not find any differences in saccade amplitudes between the scanning and verification phases, although there were reductions in the amplitude of head movements in the verification phase. There were increased fixation durations in the verification phase compared to the scanning phase for central vision, peripheral vision, and normal vision. Additionally, the verification phase involved longer fixations for the control condition compared to either central or peripheral vision conditions, although there were no differences during the scanning phase. However, the relationship between central and peripheral vision and focal and ambient modes of processing during free viewing remains to be explored. The influence of central and peripheral vision is likely different between free viewing and visual search tasks, with peripheral vision playing a substantial role in locating a target (Cornelissen et al., 2005). A resulting possibility is that differences between central and peripheral visual conditions in fixation durations and saccade amplitudes during the time course of viewing may vary between free viewing and visual search tasks.

A further factor influencing eye movement patterns is “bottom-up” factors in the form of scene content. Prior work has shown that scenes with a greater number of objects are associated with reduced saccadic amplitudes and shorter fixations (Unema et al., 2005). In work comparing scenes depicting nature with scenes depicting urban environments, there are significantly more fixations on natural scenes (Berto, Massaccesi, & Pasini, 2008), and those fixations last longer compared to urban scenes (Franěk, Šefara, Petružálek, Cabal, & Myška, 2018; Valtchanov & Ellard, 2015). Differences in scene content may also affect changes in focal and ambient modes of processing. The process of increasing fixation durations during early scene viewing takes longer to reach an asymptotic level for scenes that have more objects (Unema et al., 2005). Moreover, fixation durations and saccade amplitudes are larger for scenes depicting aerial views compared to terrestrial views, which the authors argue reflects increased dominance of ambient processing (Pannasch, Helmert, Hansen, Larson, & Loschkey, 2014). However, it remains to be determined how the presence or absence of central and peripheral vision influence the relationship between scene content and eye movement patterns.

To investigate these questions, we conducted an experiment using a gaze-contingent paradigm with free viewing of scenes presented for 20 seconds while visual access was restricted to central vision, peripheral vision, or the full visual field. Through experimentally manipulating the available visual field, we investigated the effects of central and peripheral vision on saccade amplitudes and fixation durations. We created a gaze-contingent window condition (hereafter the “central vision condition”) and a gaze-contingent scotoma condition (hereafter the “peripheral vision condition”). We explored how central and peripheral vision conditions influenced eye movement data during early and late scene processing, and in turn related to focal and ambient modes of visual processing. We also tested how fixational and saccadic patterns changed from early to late scene viewing, and whether the central vision condition, peripheral vision condition, or a control condition differed on these measures at each period. Finally, we investigated how the available visual field influenced effects of scene content on eye movements.

We carried out an initial pilot study (*n* = 16), with methodology and results reported in the Supplementary Materials. Figures from the pilot study can be found in the Supplementary Figures. Due to concerns with low power for the pilot experiment, the main experiment in this study was carried out with a larger sample size (*n* = 50) following a power analysis reported in the Methods section below.

## Hypotheses

In this experiment, the visual field was manipulated by restricting visual information using a gaze-contingent window or scotoma on each trial to create a peripheral vision condition, central vision condition, or a control condition where the full visual field was available. Following previous theory (Trevarthen, 1968), we predicted that peripheral vision should be associated with ambient visual processes, whereas central vision should be associated with focal visual processes. Because the ambient mode of visual processing involves shorter fixations and larger saccades (Pannasch et al., 2008; Unema et al., 2005), we predicted that the peripheral vision condition would involve shorter fixations and larger saccades than either the central vision or control conditions. In contrast, the central vision condition should not differ from the control condition in fixation durations, particularly during late scene viewing where focal processing should be dominant. Additionally, the central vision condition should involve shorter saccades when compared to the control condition, due to the restricted size of the visual field in this condition (Rayner, 1998).

The transition from ambient to focal processing during the time course of scene processing should involve changes in eye movements from early to late scene viewing. In the control condition, we predicted that there should be increases in fixation durations and decreases in saccade amplitude between early and late viewing intervals. Based on prior work (Pannasch & Velichkovsky, 2009), we classified fixations as ambient or focal based on their preceding saccade. We predicted that ambient fixations would have significantly smaller fixation durations compared to focal fixations. Finally, we expected to find that fixation durations and saccade amplitudes would be larger for natural scenes compared to urban scenes during the control condition.

## Methods

### Design

A within-subjects design was used, experimentally manipulating scene type (2: natural and urban) and visual condition (3: central vision, peripheral vision, and control).

### Stimuli

**Figure 1.**
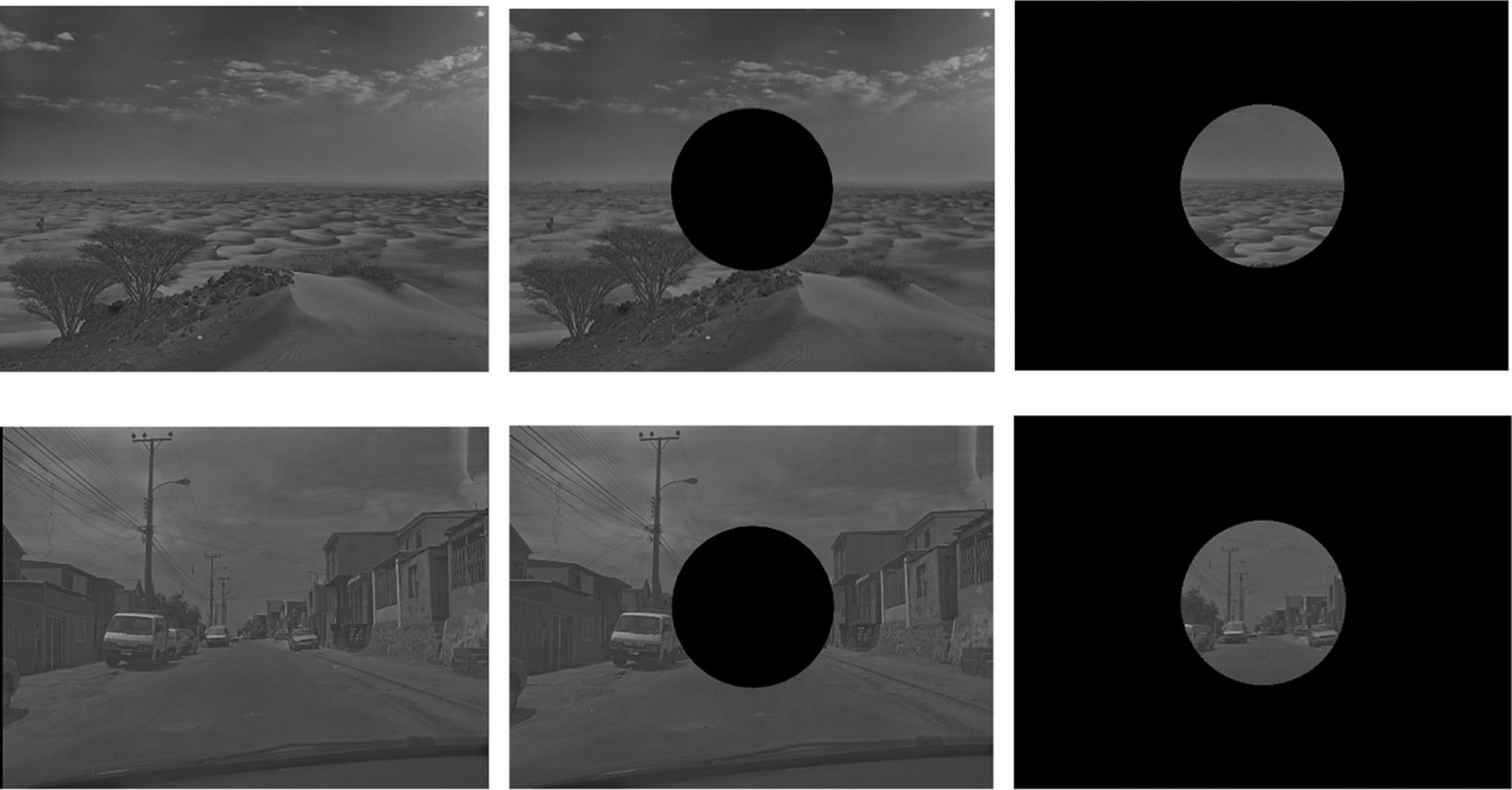
Examples from the stimulus set. Images from the top row represent a natural scene in the control, peripheral vision, and central vision conditions, while images from the bottom row represent an urban scene in the control, peripheral vision, and central vision conditions.

Ninety images of natural and urban scenes were gathered from the SUN database and from Flickr. Natural scenes consisted of forests, bodies of water, mountains, and deserts. Urban scenes consisted of city streets or skylines, building facades, and railways. The SHINE toolbox was used with MATLAB to strip scenes of color information and equalize them on low-level visual characteristics, such as spatial frequency and luminance (Willenbockel, Sadr, Fiset, Horne, Gosselin, & Tanaka, 2010). Stimuli were kept at a size of 1024 × 768 pixels, with a horizontal radius of 14.7 degrees and vertical radius of 11.2 degrees. While participants rested their head on a forehead and chinrest at a 71.5 cm distance, stimuli were presented on a CRT monitor with a refresh rate of 100 Hz. Examples of stimuli in the control, central vision, and peripheral vision conditions are displayed in Figure 1.

### Procedure

An SR Eyelink 1000 remote eye-tracking system recorded eye movements binocularly at 1000 Hz. Saccades were detected online by deflections in eye position greater than 0.1 degrees with a minimum velocity of 30 degrees/second and a minimum acceleration of 8000 degrees/second^2^, maintained for at least 4 ms. Eye position was recorded for the dominant eye of each participant. Prior to the start of the experiment, participants completed a 9-point calibration. Each trial started with the presentation of a fixation cross at the center of the screen. When participant gaze was identified at the center of the fixation cross, the scene was presented. Following this, each scene was displayed on the screen for 20 seconds. Participants were instructed to freely explore the scene and told to ensure that their gaze remained on the monitor and not outside of the scene. To implement the central and peripheral visual conditions, a gaze-contingent window or scotoma was used. In the central vision condition, only the area of the screen within the central visual field (5 degrees eccentricity) was visible, with the rest of the screen blacked out. For the peripheral vision condition, a black circle occluded the central visual field (5 degrees eccentricity). The selection of 5 degrees eccentricity as the boundary demarcating central vision from peripheral vision reflected the radius to which the fovea and parafovea extend, with peripheral vision containing the remainder of the visual field (Larson & Loschky, 2009; Rayner, 1998). To ensure that participants kept their gaze on the screen, the entire scene was occluded if participants’ gaze was located outside the boundaries of the monitor.

During the study, each participant viewed 30 scenes for each of the three visual conditions. Visual condition was subdivided by scene type, such that for each visual condition, 15 natural scenes and 15 urban scenes were presented. The order of scenes and visual condition for each scene were randomized. Two breaks were scheduled during the study after the thirtieth and sixtieth trials.

### Participants

A sample size of 50 participants was anticipated for this study. A power analysis indicated that this sample size would provide 85% power to identify an effect size of *d* = 0.50 at a significance level of 0.017 (for Bonferroni-corrected comparisons). Power was computed using the pwr package in R (Champely, Ekstrom, Dalgaard, Gill, Weibelzahl, Anandkumar, Ford, Volcic, & De Rosario, 2018). Fifty undergraduate students from the University of Waterloo with normal or corrected-to-normal vision participated in the experiment. Eye movement recordings from eight participants were incomplete due to technical issues. Those participants were thus excluded from the final sample of 42 participants. A subsequent power analysis indicated that a sample size of 42 provided 77% power to identify an effect size of *d* = 0.50 at a significance level of 0.017. Participants gave their written, informed consent prior to the experiment. The study had been approved by a University of Waterloo ethics committee.

### Data analysis

Data analysis was carried out using R (R Core Team, 2021). Blinks were identified as periods in which pupil information was missing. Fixations or saccades that started before the scene was initially presented were removed. Three thousand five hundred fifty-three (3553) fixations and 203 saccades were removed under this criterion, representing 1.9% of the data set. Saccades or fixations occurring within a 100 ms interval of a blink were discarded. This resulted in the removal of 43,755 fixations and 26,645 saccades, representing 19.9% of the data set. Any fixations longer than 1000 ms or shorter than 80 ms were removed. This resulted in the removal of 8253 fixations, representing 3.5% of the data set. Trials where the total blink time was equal to or greater than 4 seconds (20% of the trial length) were excluded from the data set. This resulted in the removal of 266 trials, representing 7.0% of the data set. Due to these criteria, a total of 32.4% of the data set was excluded from analyses. A Greenhouse-Geisser correction was applied for repeated-measures ANOVAs where violations of sphericity were present. The data set can be found on an Open Science Framework (OSF) repository (https://osf.io/nw6ym/).

## Results

### Fixation durations

**Figure 2.**
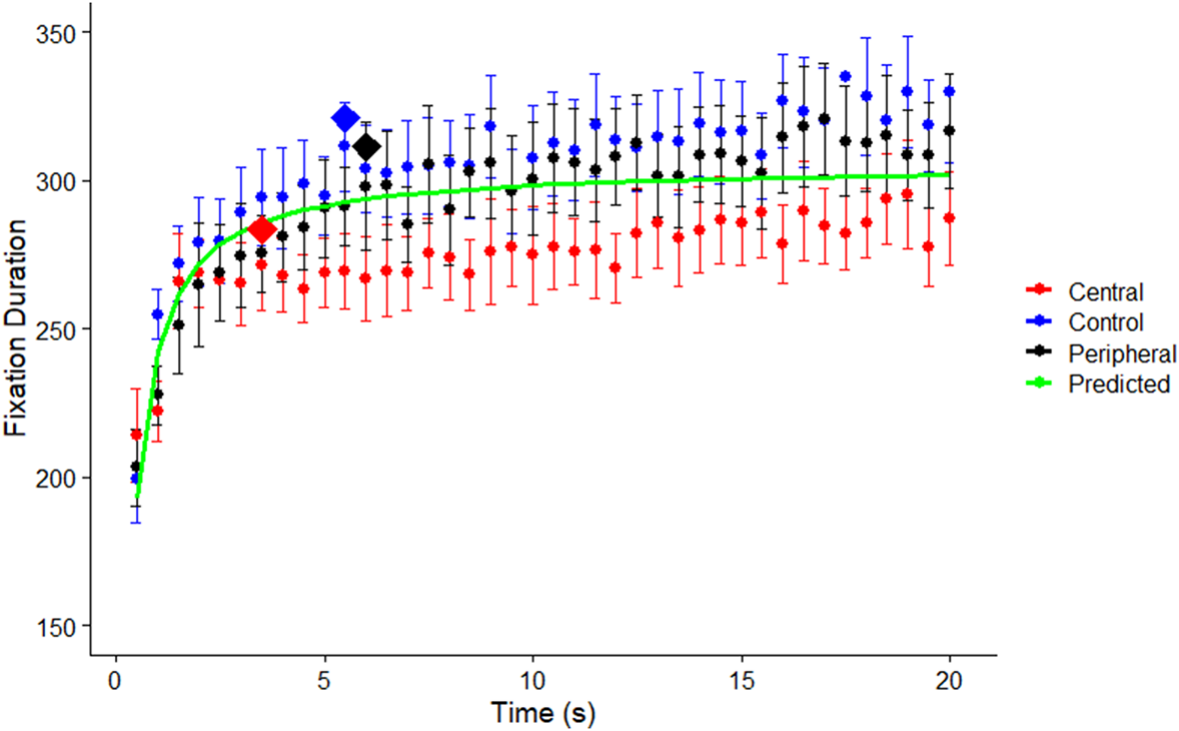
Mean fixation durations for the central vision, peripheral vision, and control conditions calculated for each 500 ms time interval during scene viewing. Error bars represent 95% confidence intervals for each condition. Points represented via diamonds indicate the asymptotic value for each visual condition (y-axis) and the time at which asymptote was reached (x-axis). Predicted values for each time interval were from a nonlinear regression across all visual conditions.

A nonlinear regression analysis evaluated the time course of fixation durations during scene viewing. Viewing periods for each scene were parceled into 500 ms bins, with mean fixation durations calculated for each of those bins. ^1^Figure 2 depicts changes in mean fixation durations calculated for every 500 ms time interval during scene viewing in each of the three visual conditions, along with predicted values at each interval based on the nonlinear regression. The model consisted of the following equation:

(1) $$\begin{array}{c} \mathrm{fd}=b*e^{a/t}\; \end{array}$$

In this equation, *b* represents the asymptote, whereas *a* represents the acceleration rate. The regression function calculated to best fit the data was as follows:

(2) $$\begin{array}{c} \mathrm{fd}=305.3*e^{-0.23/t}\; \end{array}$$

The asymptotic value was 305.3 ms (95% confidence intervals [CIs] = 287.1 to 323.6 ms). As expected, fixation duration increased rapidly during the first few seconds of viewing. A condition was defined as having reached asymptote at the first point where the asymptote was within when the 95% CI of the mean value for a 500 ms interval.

Subsequent analyses indicated that whereas this increase in fixation duration was identified across all visual conditions, the magnitude of the asymptote and length of time required to reach the asymptote were different for each condition. For the central vision condition, the asymptote was 283.5 ms (95% CIs = 280.5 to 286.4 ms), reached at 3.5 seconds. For the peripheral vision condition, the asymptote was 311.4 ms (95% CIs = 308.4 to 314.5 ms), reached at 6 seconds. For the control condition, the asymptote was 321.2 ms (95% CIs = 318.1 to 324.2 ms), reached at 5.5 seconds.

### Saccade amplitudes

As with fixation durations, viewing periods for each scene were parceled into 500 ms bins, with mean saccade amplitudes calculated for each of those bins. There was an increase in saccade amplitudes from bins one and two reaching a peak during bin three (at 1.5 seconds), followed by a subsequent decline for the next few seconds and subsequent plateau. To identify the point at which saccade amplitudes reached asymptote, the first two bins were excluded from the nonlinear regression in order to be able to use the same nonlinear regression model as in (Equation 1). The model was as follows:

(3) $$\begin{array}{c} \mathrm{sa}=b*e^{a/t}\; \end{array}$$

With *b* representing the asymptote and *a* representing the acceleration rate, the regression function calculated was:

(4) $$\begin{array}{c} \mathrm{sa}=5.7*e^{0.4/t}\; \end{array}$$

**Figure 3.**
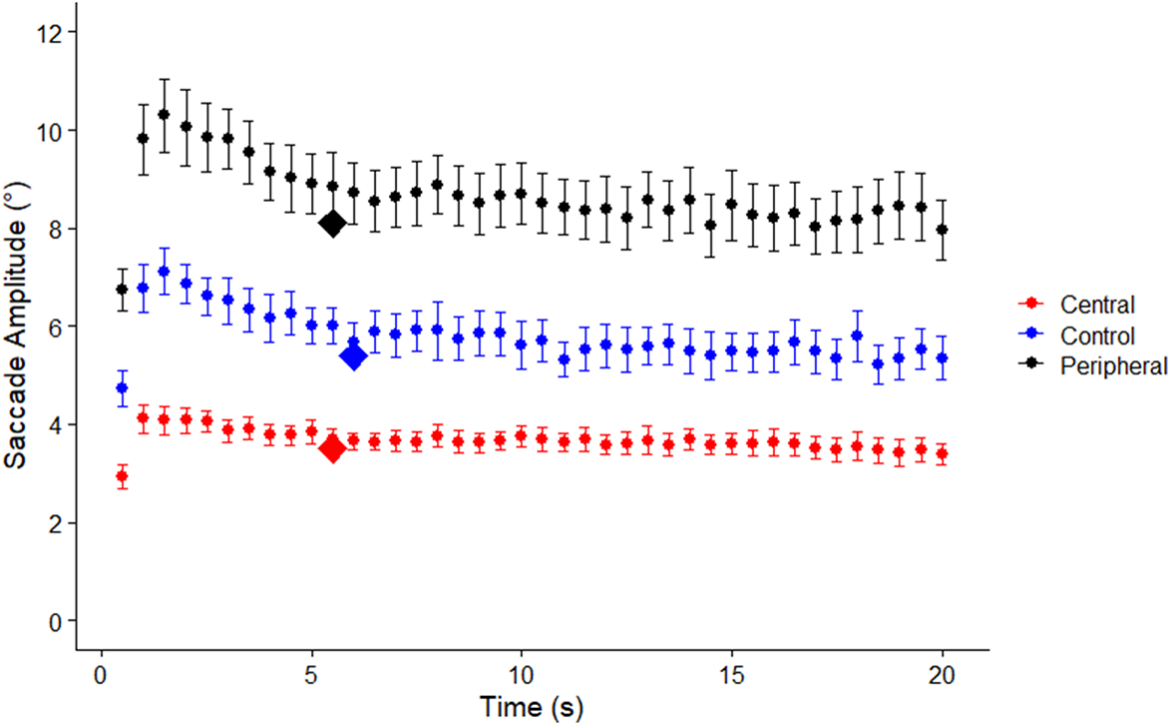
Mean saccade amplitudes for the central vision, peripheral vision, and control conditions calculated for each 500 ms time interval during scene viewing. Error bars represent 95% confidence intervals for each condition. Points represented via diamonds indicate the asymptotic value for each visual condition and the time at which asymptote was reached.

The asymptotic value for this model was 5.7 degrees (95% CIs = 3.5 degrees to 7.9 degrees). As expected, saccade amplitudes declined rapidly after the first second of viewing, an effect found in all visual conditions. Figure 3 depicts changes in mean saccade amplitudes calculated for every 500 ms time interval during scene viewing in each of the three visual conditions.

As with fixation durations, the magnitude of the asymptote for saccade amplitudes and length of time required to reach the asymptote were different for each condition. For the central vision condition, the asymptote was 3.5 degrees (95% CIs = 3.5 to 3.6 degrees], reached at 5.5 seconds. For the peripheral vision condition, the asymptote was 8.1 degrees (95% CIs = 8.1 to 8.2 degrees), reached at 5.5 seconds. For the control condition, the asymptote was 5.4 degrees (95% CIs = 5.3 to 5.5 degrees), reached at 6 seconds.

### Visual field, scene type, and fixations/saccades

**Figure 4.**
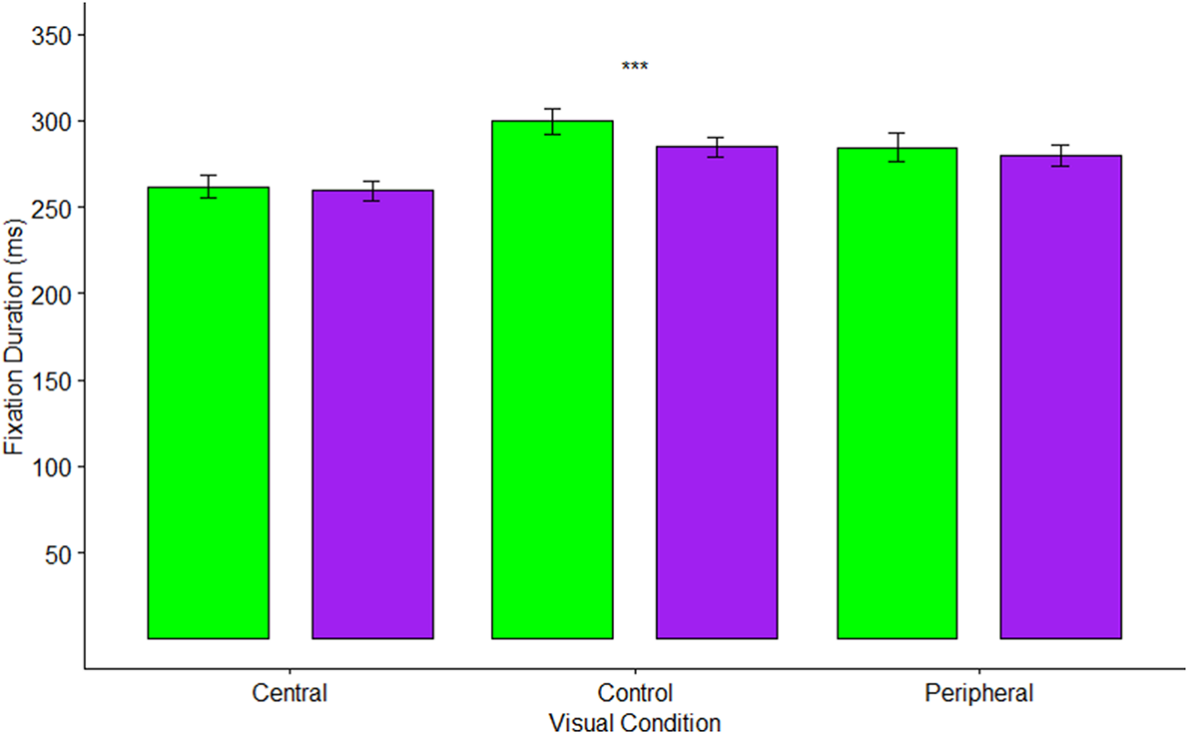
Mean fixation durations by visual condition and scene type. Green bars indicate natural scenes, while purple bars indicate urban scenes. Error bars indicate one standard error. *** represents *p* < 0.0003.

A repeated-measures ANOVA tested the effects of visual condition (central, control, and peripheral) and scene type (natural and urban) on fixation durations. Figure 4 depicts mean fixation durations by scene type and visual condition. There were significant effects of visual condition (*F*(2,82) = 27.42, *p* < 0.001, η^2^_p_ = 0.40), and scene type (*F*(1,41) = 14.37, *p* < 0.001, η^2^_p_ = 0.26) on fixation duration, qualified by a significant visual condition times scene interaction (*F*(2,82) = 8.49, *p* < 0.001, η^2^_p_ = 0.17). Bonferroni-corrected planned comparisons (α = 0.017) indicated that fixation durations were longer in the control condition (*M* = 292.1 ms) when compared to the central vision condition *(M* = 260.5 ms), *t*(41) = 7.07, *p* < 0.001, but not when compared to the peripheral vision condition (*M* = 282.0 ms), *t*(41) = 2.36, *p* = 0.023. This was inconsistent with predictions, as we had expected fixation durations to be longer in the control condition compared to the peripheral vision condition, but not different between the control and central vision conditions. We found that the peripheral vision condition involved longer fixations than the central vision condition (*t*(41) = 4.95, *p* < 0.001), an effect which was in the opposite direction to what was initially predicted.

Simple effects tests found a significant effect of scene type on fixation duration for the control condition (*F*(1,41) = 22.80, *p* < 0.001, η^2^_p_ = 0.36). As hypothesized, natural scenes (*M* = 299.5 ms) involved longer fixations than urban scenes (*M* = 284.5 ms). There were no effects of scene type for either the central vision (*Ms* = 261.8, 259.2 ms), *F*(1,41) = 1.62, *p* = 0.21, η^2^_p_ = 0.04, or peripheral vision conditions (*Ms* = 284.5, 279.7 ms), *F*(1,41) = 2.90, *p* = 0.10, η^2^_p_ = 0.07.

There were significant effects of visual condition (*F*(1.35,55.37) = 248.54, *p* < 0.001, η^2^_p_ = 0.86), scene type (*F*(1,41) = 12.60, *p* < 0.001, η^2^_p_ = 0.24), and a visual condition x scene interaction (*F*(2,82) = 15.98, *p* < 0.001, η^2^_p_ = 0.28) on saccade amplitudes. Consistent with hypotheses, planned comparisons (α = 0.017) revealed that saccade amplitudes were significantly larger in the peripheral vision condition (*M* = 8.6 degrees) when compared to either the central vision (*M* = 3.7 degrees) or control conditions (*M* = 5.8 degrees). As predicted, there were significantly larger saccades in the control condition when compared to the central vision condition (all *p* values < 0.001).

**Figure 5.**
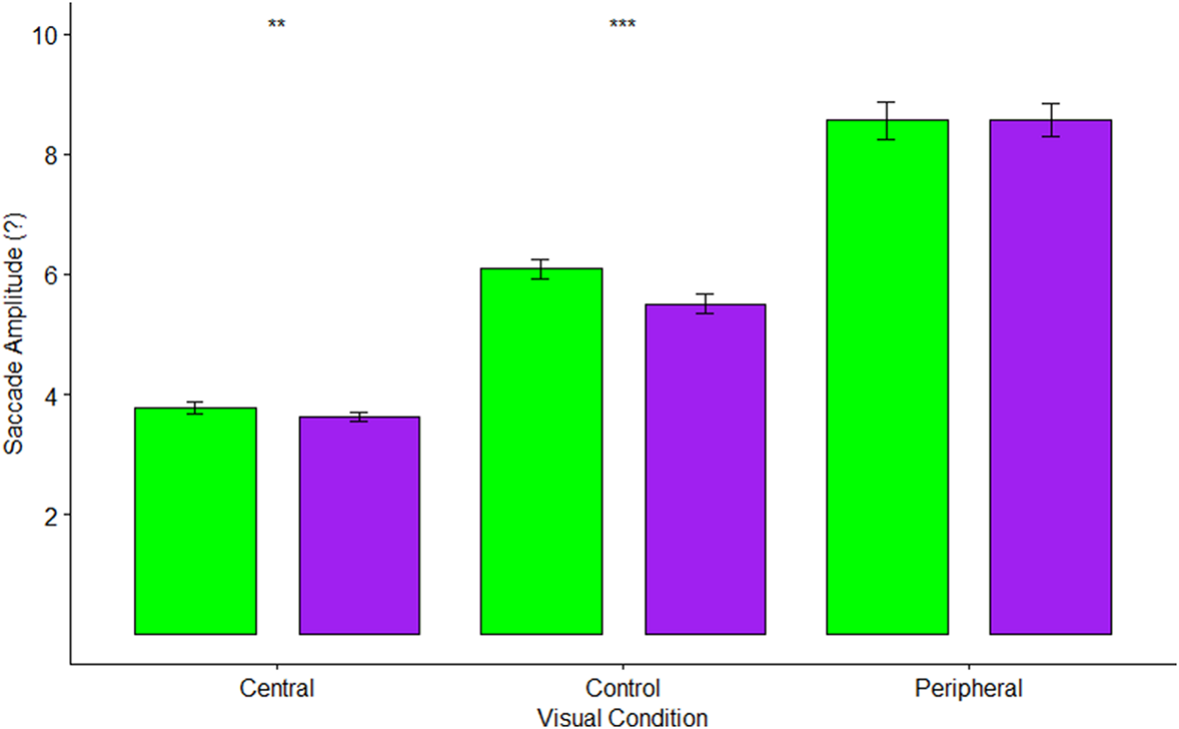
Mean saccade amplitudes by visual condition and scene type. Green bars indicate natural scenes, while purple bars indicate urban scenes. Error bars indicate one standard error. ** Represents *p* < 0.003, *** represents *p* < 0.0003.

Figure 5 depicts mean saccade amplitudes by scene type and visual condition. As predicted, simple effects tests (α = 0.017) indicated that saccade amplitudes were significantly larger for natural scenes than urban scenes for the control condition (*M*s = 6.1 degrees, 5.5 degrees), *F*(1,41) = 30.90, *p* < 0.001, η^2^_p_ = 0.43. For central vision, saccade amplitudes were also significantly larger for natural scenes (*M* = 3.8 degrees) when compared to urban scenes (*M* = 3.6 degrees), *F*(1,41) = 10.80, *p* = 0.002, η^2^_p_ = 0.21. There were no differences for peripheral vision (*M*s = 8.6 degrees, 8.6 degrees), *F*(1,41) = 0.00, *p* = 0.96, η^2^_p_ = 0.00.

### Visual field and early/late processing

**Figure 6.**
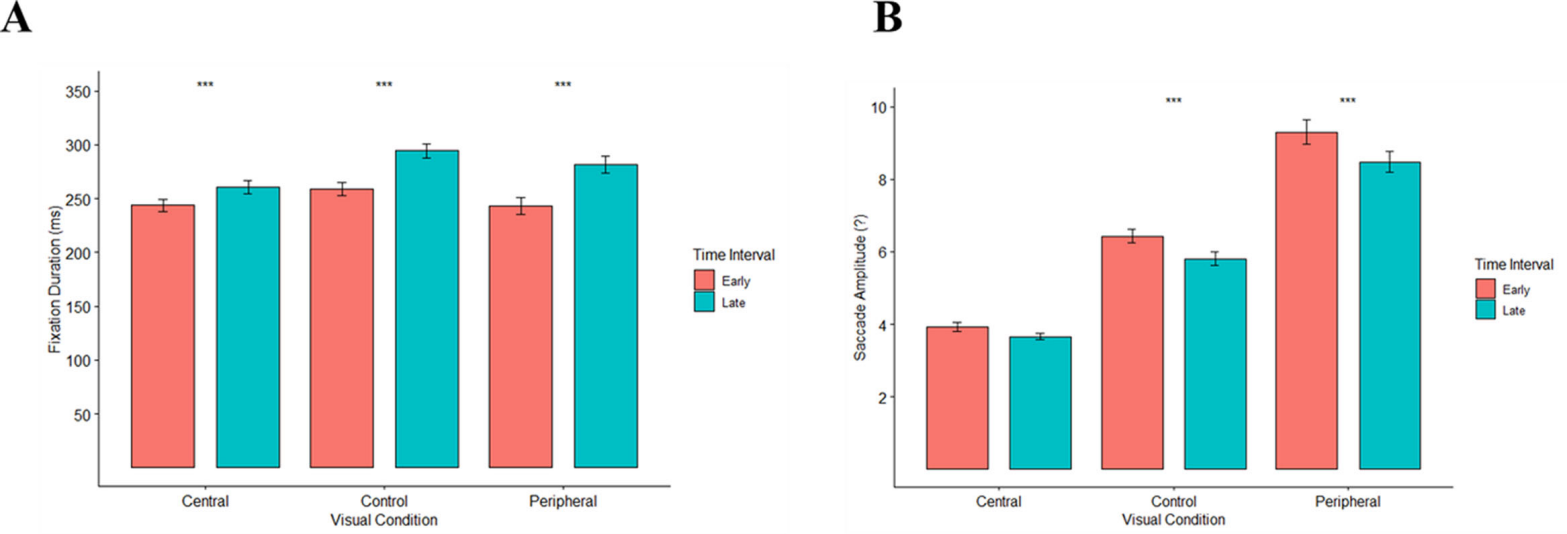
Mean fixation durations (A) and saccade amplitudes (B) by visual condition and time interval. Red bars indicate early time intervals (0-2 seconds), whereas the blue bars indicate late time intervals (6-8 seconds) during scene viewing. Comparisons represent pairwise comparisons between early and late time intervals for each visual condition. Error bars indicate one standard error. *** Indicates *p* < 0.0003.

A two-way repeated-measures ANOVA tested the effects of time interval (2: early and late) or visual condition (3: control, central, and peripheral) on fixation durations. Early time intervals consisted of the first 2 seconds of scene presentation following Pannasch et al. (2008). Data from this study (see Figure 2) indicated that asymptotic fixation durations for the peripheral condition occurred at 6 seconds, whereas the control vision condition (5.5 seconds) and central vision conditions (3.5 seconds) reached asymptotic values earlier. Thus, we defined late time intervals in this study using a 2-second window between 6 and 8 seconds after scene presentation, as asymptote would have been reached by this point in all visual conditions. We compared fixation and saccade responses during this late time interval with the early time interval (0-2 seconds). Figure 6A depicts mean fixation durations in early and late time intervals for each of the three visual conditions. There was a significant effect of visual condition (*F*(2,82) = 12.51, *p* < 0.001, η^2^_p_ = 0.23) and a significant effect of time interval (*F*(1,41) = 77.47, *p* < 0.001, η^2^_p_ = 0.65), qualified by a significant visual condition times time interaction (*F*(2,82) = 6.25, *p* = 0.003, η^2^_p_ = 0.13). Simple effects tests (Bonferroni-corrected α = 0.017) found significant effects of time interval on fixation duration for each of the three visual conditions (*F*s(1,41) > 16, *p* values < 0.001, η^2^_p_s > 0.29). In central vision (*M*s = 243.6, 260.6 ms), peripheral vision (*M*s = 243.2, 281.5 ms), and the control condition (*M*s = 259.2, 294.1 ms), fixation durations were significantly shorter within early time intervals when compared to late intervals. This pattern was consistent with predictions, as we had expected fixation durations to increase from early to late time intervals.

Simple effects tests (Bonferroni-corrected α = 0.025) indicated significant effects of visual condition on fixation durations for both early (*F*(2,82) = 4.65, *p* = 0.024, η^2^_p_ = 0.10) and late time intervals (*F*(2,82) = 17.40, *p* < 0.001, η^2^_p_ = 0.29). For early time intervals, planned contrasts (α = 0.017) indicated that fixation durations in the control condition were significantly longer than the peripheral vision condition (*t*(41) = 2.57, *p* = 0.014), but not different from the central vision condition (*t*(41) = 2.46, *p* = 0.018). This was consistent with predictions, as it was expected that the control condition would involve longer fixations than the peripheral vision condition during early time intervals. Surprisingly, the peripheral vision and central vision conditions did not differ from each other during the early time interval (*t*(41) = 0.08, *p* = 0.94). For the late time interval (α = 0.017), the control condition involved significantly longer fixation durations than the central vision condition (*t*(41) = 5.99, *p* < 0.001), but not when compared to the peripheral vision condition (*t*(41) = 2.08, *p* = 0.04). There were also significantly longer fixations for the peripheral vision condition when compared to the central vision condition for this interval (*t*(41) = 3.60, *p* < 0.001). Results for the late time interval were almost entirely opposite to predictions, as we had expected the control and central vision conditions to involve similar fixation durations, and the peripheral visual condition to have shorter fixation durations compared to the other two conditions. Instead, we found that the central vision condition had the shortest fixation durations during the late time interval.

For saccade amplitudes, a repeated-measures ANOVA found a main effect of visual condition (*F*(1.38,56.7) = 237.002, *p* < 0.001, η^2^_p_ = 0.85) and a main effect of time interval (*F*(1,41) = 31.46, *p* < 0.001, η^2^_p_ = 0.43), qualified by a significant interaction (*F*(2,82) = 5.31, *p* = 0.007, η^2^_p_ = 0.12). Simple effects tests (α = 0.017) indicated significant effects of time interval for the peripheral vision (*F*(1,41) = 21.4, *p* < 0.001, η^2^_p_ = 0.34) and the control conditions (*F*(1,41) = 24.1, *p* < 0.001, η^2^_p_ = 0.37), whereas the effect for the central vision condition was not significant (*F*(1,41) = 5.82, *p* = 0.020, η^2^_p_ = 0.13). As predicted, saccades during early time intervals were significantly shorter than saccades during late time intervals for the control and peripheral visual conditions, whereas the effect for the central vision condition was in the same direction but non-significant post-Bonferroni correction. Figure 6B depicts mean saccade amplitudes in early and late time intervals for each of the three visual conditions. Planned contrasts indicated that, as predicted, during the early time interval, the peripheral vision condition (*M* = 9.3 degrees) involved larger saccade amplitudes than the control condition (*M* = 6.4 degrees), which in turn were larger than the central vision condition (*M* = 3.9 degrees), all *p* values < 0.001. An identical pattern occurred for the late time interval, with the peripheral vision condition (*M* = 8.5 degrees) involving larger amplitudes than the control condition (*M* = 5.8 degrees), which in turn were larger than the central vision condition (*M* = 3.7 degrees), all *p* values < 0.001. These results were consistent with the hypothesis that the peripheral vision condition should involve larger saccade amplitudes than the control condition, which in turn should involve larger saccades than the central vision condition, due to differences in the information available to the visual system for saccade planning in the three conditions. Furthermore, it is consistent with the hypothesis that such differences in saccade amplitudes between the three conditions should be accentuated during the early time interval, when the ambient mode of vision is active.

### Focal and ambient fixations

**Figure 7.**
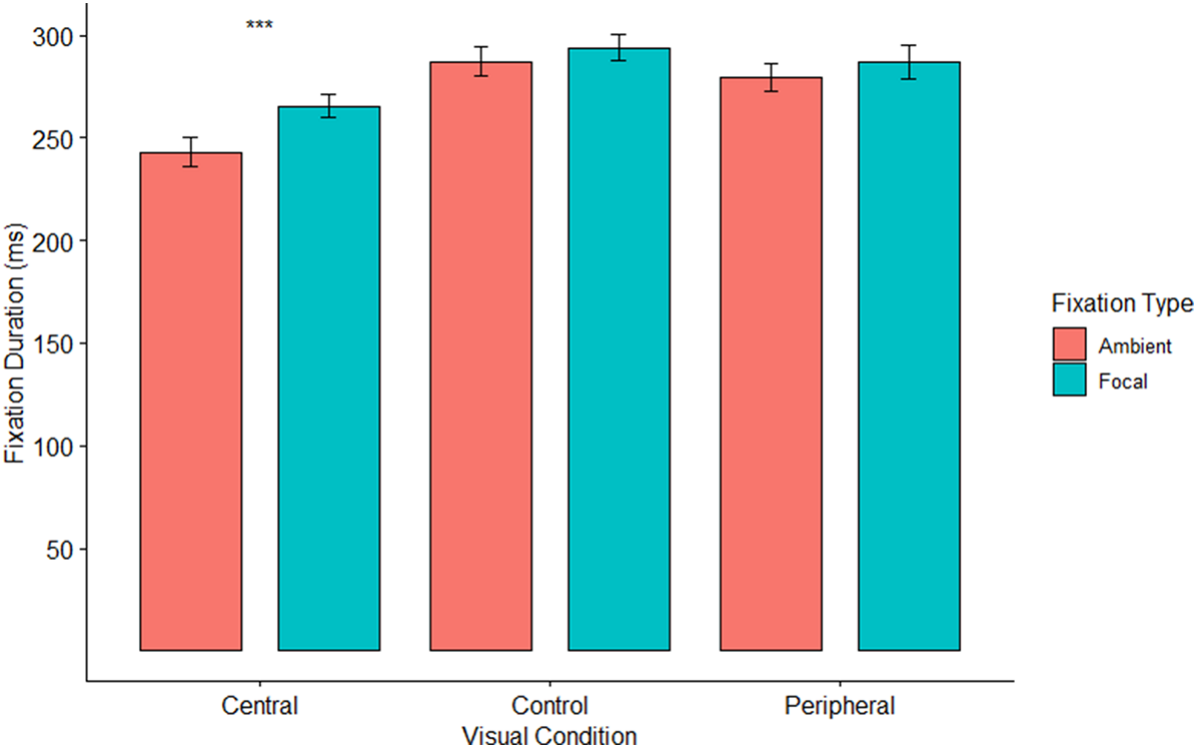
Mean fixation durations by visual condition and fixation type. Red bars indicate mean ambient fixations, while blue bars indicate mean focal fixations. Error bars indicate one standard error. *** Represents *p* < 0.0003.

Fixations were classified as ambient or focal on the basis of their preceding saccade, as per Pannasch and Velichkovsky (2009). Ambient fixations were preceded by a saccade with an amplitude >5 degrees, and focal fixations were preceded by a saccade with an amplitude <5 degrees. There were significant effects of visual condition (*F*(2,82) = 33.07, *p* < 0.001, η^2^_p_ = 0.45) and fixation type (*F*(1,41) = 34.29, *p* < 0.001, η^2^_p_ = 0.46) on fixation durations, as shown in Figure 7. As predicted, ambient fixation durations (*M* = 269.9 ms) were shorter than focal fixation durations (*M* = 282.1 ms). These effects were qualified by a significant interaction (*F*(1.75,71.8) = 9.72, *p* < 0.001, η^2^_p_ = 0.19). Simple effects tests indicated that the effect of fixation type on fixation duration was significant for the central vision condition (*F*(1,41) = 68.90, *p* < 0.001, η^2^_p_ = 0.63), but not significant for the peripheral vision (*F*(1,41) = 4.33, *p* = 0.044, η^2^_p_ = 0.10) or control conditions (*F*(1,41) = 5.08, *p* = 0.030, η^2^_p_ = 0.11) due to the lower alpha level (α = 0.017). The directions of the effects were consistent with predictions, with fixation durations being shorter for ambient fixations relative to focal fixations for the central vision (*M*s = 243.1, 265.5 ms), peripheral vision (*M*s = 279.5, 287.0 ms), and control conditions (*M*s = 287.2, 293.9 ms).

## Discussion

The purpose of this study was to identify (1) the influence of central and peripheral vision on fixation durations and saccade amplitudes, (2) the effect of the visual field on changes in eye movements throughout scene viewing from early to late visual processing, (3) the role of central and peripheral vision during focal and ambient processing, and (4) whether differences in scene content result in differing eye movement patterns. We found that fixation durations and saccade amplitudes when viewing scenes were significantly influenced by differences in the available visual field. Contrary to predictions, fixation durations were longer in the control condition than they were in the central vision condition, instead of being equal as expected. Surprisingly, fixation durations were not significantly larger in the control condition compared to the peripheral vision condition, post-Bonferroni correction. Additionally, fixation durations were significantly larger in the peripheral vision condition when compared to the central vision condition. Due to reduced power, the pilot study (see supplementary materials) only found a significant difference in fixation durations between the control and central vision conditions, which was in the same direction as the main experiment in this study. In addition to main effects of visual condition, time interval had a significant impact on fixation durations. Fixation durations increased significantly from early to late time intervals in every visual condition. In prior research, similar effects have been found for scene processing under normal visual conditions (Antes, 1974; Castelhano, Mack, & Henderson, 2009; Irwin & Zelinsky, 2002; Yarbus, 1967). This pattern is also consistent with other work analyzing changes in fixation durations from early to late visual processing manipulating scene familiarity by presenting terrestrial and aerial views of scenes (Pannasch et al., 2014). Increases in fixation durations during the time course of scene processing are theorized to reflect a transition from ambient to focal processing, resulting in longer fixations on the most salient elements of a scene, rather than exploratory visual scanning (Pannasch et al., 2008; Unema et al., 2005). It is thus significant that fixation durations reliably increased over time for scenes viewed through peripheral vision. The absence of foveal information did not prevent the ambient-to-focal transition in visual processing of scenes. Although a lack of foveal information may impair the ability to gather useful information out of longer fixations, this did not prevent an increase in fixation durations. In fact, the current study found that peripheral vision involves longer fixation durations during late scene processing than central vision. It is likely that the ambient-to-focal transition is a built-in process in the visual system that cannot be disrupted simply by a 1-hour experiment, particularly when there are three separate visual conditions involved. In experiments involving simulated retinal loss, the creation of a preferred retinal locus for fixation tends to involve hours’ worth of training over multiple days (Kwon, Nandy, & Tjan, 2013; Maniglia, Jogin, Visscher, & Seitz, 2020). One possibility is that participants in our experiment were making long fixations in the periphery during late processing through executing innate patterns of visual exploration and examination. However, it is unclear how much additional or meaningful information participants were able to gain in the absence of foveal vision through those long fixations in the peripheral condition.

The difference in fixation durations during late scene processing drove a broader effect in which fixations in the peripheral vision condition were longer than fixations in the central vision condition. This pattern is quite surprising, considering that late scene processing involves visual tasks that would seem to be strongly associated with central vision. Focusing on specific objects or regions of interest and making long fixations to gather detailed information are all tasks that rely on the fovea. It is difficult to attribute the relative length of fixations to increases of task difficulty. Durations of peripheral fixations during late viewing were largely similar to the control condition, even though in normal vision the task of exploring a scene was easier due to greater availability of information. Moreover, the negative impact of the absence of foveal vision must have been exacerbated by the fact that there is cortical magnification of the fovea in visual cortex (Cowey & Rolls, 1974; Curcio & Sloan, 1992; Duncan & Boynton, 2003). However, studies invoking task difficulty as the primary explanation often find an increase in fixation durations when vision is masked in the center or periphery when compared to normal vision (Cornelissen et al., 2005; Nuthmann, 2014). Although there are some experiments finding decreased fixation durations for peripheral vision compared to control (David et al., 2019; Henderson et al., 1997), they did not find similar decreases for central vision compared to peripheral vision. One explanation for why fixation durations in the central vision condition may be shorter is because less information is present in each fixation. Limiting visual content may mean that less time is required for available visual content to be processed in each fixation. This may also explain why the length of time required to reach asymptote for fixation durations was smaller for the central vision (3.5 s) condition compared to either peripheral vision (6 seconds) or the control condition (5.5 seconds). Reaching the asymptotic value may take less time if the asymptotic fixation duration is relatively smaller in the central vision condition and closer to fixation durations during early time intervals. However, one challenge is that fixation durations during early time intervals should be smaller for the central vision condition compared to the peripheral vision condition. In this study, we did not observe any such difference between the central and peripheral vision conditions in fixation durations during early viewing, finding a null effect instead. In the pilot study, there were no significant differences between any of the visual conditions during the early time interval, though this is likely explained by low power.

The results in our experiments were also unique in terms of the differences found during early and late visual processing. One VR study investigating visual search found no effects of visual field on fixation durations during the scanning phase, and that fixations in the peripheral vision condition were shorter than the control condition during the verification phase (David et al., 2020). In contrast, the main experiment in our study found significant effects during early visual processing, with fixations longer in the control condition compared to the peripheral vision condition. Some possible explanations for these variations include differences in the task (visual search vs. free viewing), demarcation of central and peripheral vision (6 degrees in their study versus 5 degrees in this experiment), and presentation mode (VR versus desktop presentation). An alternative possibility is that changes in fixation durations between visual conditions and time intervals in our experiment point to contextual information provided by the visual periphery playing a substantial role in later fixations. Whereas central vision was excellent at focusing on objects of interest and identifying specific details, that information may have been challenging to interpret and utilize with a lack of peripheral context. As a result, fixations during late time intervals during the central vision condition may have been cut short relative to the peripheral vision and control conditions, allowing for more useful exploration of the scene. In contrast, the peripheral vision condition involved sufficient information to grasp the context, even though foveal information was lacking.

This study found substantial effects of visual condition on saccade amplitudes. The peripheral vision condition involved significantly larger saccade amplitudes than the control condition, while the control condition involved significantly larger saccade amplitudes than the central vision condition. This effect was likely caused by the need to maximize useful visual information in each fixation. In the peripheral vision condition, more saccades were made to regions outside of the scotoma, whereas in the central vision condition, more saccades were made within the central window. The saccade amplitude required to fixate on a region of interest that one can currently see was much higher for participants in the peripheral vision condition. In the central vision condition, making a subsequent fixation on some visual element currently of interest would involve making a second fixation within the visible central window, and thus involved a much shorter saccade amplitude. The result for the central vision condition is in line with prior studies showing that restricting visual information to central vision results in reduced saccade amplitudes (Shioiri & Ikeda, 1989) and that the size of saccade amplitudes increases with the size of the perceptual window (Loschky & McConkie, 2002; Rayner et al., 1981). Similarly, increased saccadic amplitudes when vision was limited to the periphery is consistent with prior research (Laubrock et al., 2013; van Diepen, 2002). There were some differences between the main experiment and the pilot study in how saccade amplitudes changed during the time course of scene processing. In the main experiment, saccade amplitudes for the control and peripheral visual conditions decreased significantly from early to late time intervals, whereas effects for the central vision condition were in the same direction but not significant. Moreover, the length of time required to reach asymptote for saccade amplitudes did not widely differ between the three visual conditions, with asymptote reached at 5.5 seconds for the central and peripheral vision conditions, and at 6 seconds for the control condition. This pattern of results contrasts with the David et al. (2020) study in which there were no differences in saccade amplitudes between scanning and verification phases, but only differences in head movements. In the pilot study, there were no significant decreases in saccade amplitude between early and late processing in any of the conditions. Although these differences were likely due to reduced power in the pilot, a competing explanation describing results for the central vision condition is the fact that central vision is the least likely to involve larger exploratory saccades during early visual processing. Decreases in saccade amplitudes over the time course of scene processing may have been smaller for the central visual condition when compared to the peripheral vision and control conditions, where initial exploratory eye movements provided much more useful information and involve larger saccades. If initial saccades in the central vision condition were very small to begin with, there may have been less room for those saccades to have even shorter amplitudes.

The experiment also indicated differences by fixation type on fixation duration. As predicted, focal fixations were significantly longer when compared to ambient fixations. These results are consistent with prior work delineating fixations as ambient or focal, which argue that ambient fixations are shorter in length (Pannasch et al., 2008). Because fixation type was determined by the size of the preceding saccade, ambient fixations were much more prevalent in early visual processing, whereas focal fixations were more prevalent in late visual processing, where there were much smaller saccades. Surprisingly, the effect of fixation type on fixation duration was mainly driven by the central vision condition. Differences between ambient and focal fixation durations were not significant in the control or peripheral vision conditions. Although this may have been due to the lower alpha level (α = 0.017) for the simple effects test (*p* values = 0.03, 0.044), the pilot study also found a similar pattern, with significant differences only in the central vision condition. These results are consistent with prior work delineating fixations as ambient or focal, which argue that ambient fixations are shorter in length and more frequent during early visual processing (Pannasch et al., 2008).

For processing of natural and urban scenes, both the main experiment and pilot study indicated that in the control condition, natural scenes involved significantly longer fixations and larger saccade amplitudes compared to urban scenes. These results are consistent with previous studies investigating the influence of natural versus urban scene content on fixation durations (Franek et al., 2018; Valtchanov & Ellard, 2015), and expand on them to show differences in mean saccade amplitudes by scene content. For the central vision condition, saccade amplitudes were also significantly larger for natural scenes when compared to urban scenes, although there were no effects of scene type for fixation durations. For the peripheral vision condition, there were no differences in fixation durations or saccade amplitudes by scene type. One implication of these results is that fixation and saccade patterns for the peripheral vision condition in this study do not appear to be particularly responsive to scene content. One possibility is that the lack of any effect of scene type on eye movements indicates that for the peripheral vision condition, eye movement patterns were largely a result of top-down visual processes. Fixations and saccades may have been influenced more strongly by strategies to maximally explore each scene, rather than reacting to scene content. In contrast, the effect of scene type on saccade amplitudes for the central vision condition indicates a potentially greater influence of scene content on visual exploration. A second possibility is that scene type had little effect on fixation durations in the central or peripheral vision conditions due to the inability of either peripheral or central vision to fully process content information in the absence of the other. Fixation durations may have been similar between natural and urban scenes in the central vision condition because the lack of contextual information made it challenging to fully grasp information from each fixation. In the peripheral vision condition, the lack of information from the fovea made it much more challenging to focus on items of interest within a scene. To address the question of how central and peripheral vision process scene content, further work should explore how judgments of scenes are influenced by the visual field, and how fixational patterns relate to the ability to form these decisions.

Another factor explaining both fixational patterns is the type of task involved. This study exclusively used a free-viewing procedure. Work involving visual search finds dissimilar results in terms of differences in fixation durations between central, peripheral, and full vision conditions during early and late time intervals (David et al., 2020). Other work suggests that task type may impact fixation durations and saccade amplitudes (Cronin, Hall, Goold, Hayes, & Henderson, 2020; Mills, Hollingworth, van der Stigchel, Hoffman, & Dodd, 2011). Task type may also influence the transition from ambient to focal processing, through influencing the rate of change of fixation durations (Mills et al., 2011), although other studies indicate that effects may be limited to other measures such as aggregate fixation time or number of fixations (Castelhano et al., 2009).

## Conclusions

Restricting visual information to either the central or peripheral visual field has substantial effects on fixation durations and saccade amplitudes during scene processing. Although previous studies show mixed effects of differences between central vision, peripheral vision, and full vision on fixation durations, we found that fixation durations are consistently shorter when vision is restricted to central vision, compared to normal vision. We demonstrated that fixation durations were significantly higher for the peripheral vision condition than the central vision condition during late visual processing, and were similar to full vision. We also showed that the transition from ambient to focal visual processing characterized by increased fixation durations and decreased saccade amplitudes will occur even when people are restricted to central or peripheral vision conditions. Future work should not only investigate the functions of central and peripheral vision in isolation, but also how they interact throughout the time course of scene processing.

## Supplementary Material

Supplement 1

Supplement 2
