# Supplementary material for: How central and peripheral vision influence focal and ambient processing during scene viewing
Source: J Vis. 2022 Nov 2;22(12):4. doi: 10.1167/jov.22.12.4 (PMC9639699; doi:10.1167/jov.22.12.4)
Supplement: Supplement 1 [file jovi-22-12-4_s001.pdf]

Supplementary Figures

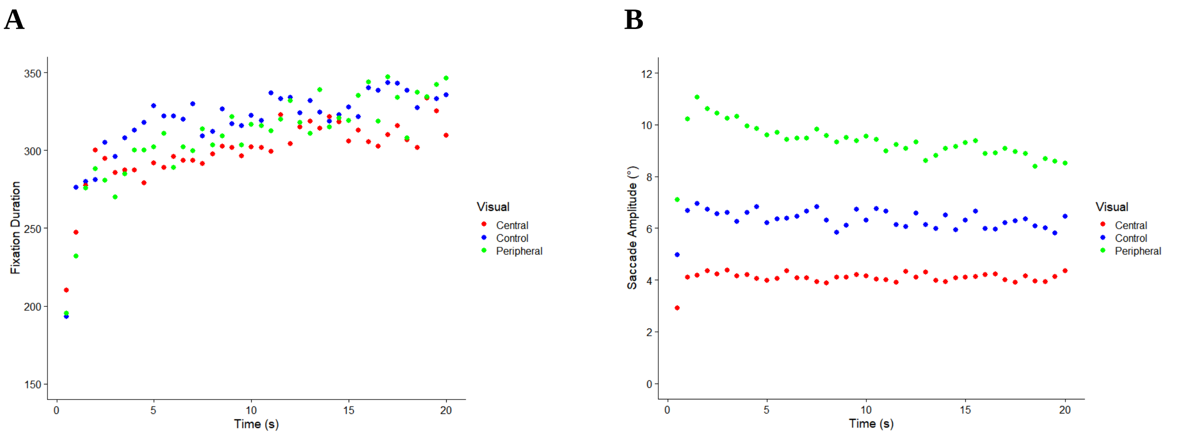

Figure 1. Mean fixation durations (A) and saccade amplitudes (B) for the central vision, peripheral vision, and the control conditions calculated for each 500 ms time interval during scene viewing.

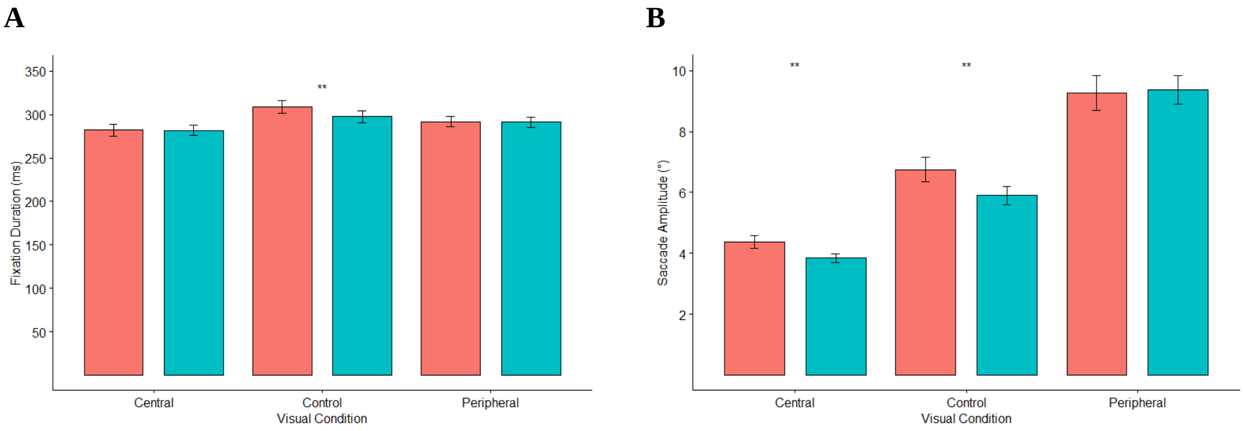

Figure 2. Mean fixation durations (A) and saccade amplitudes (B) by visual condition and scene type. Red bars indicate natural scenes, while blue bars indicate urban scenes. Error bars indicate one standard error. ** indicates p < .003.

A **B**

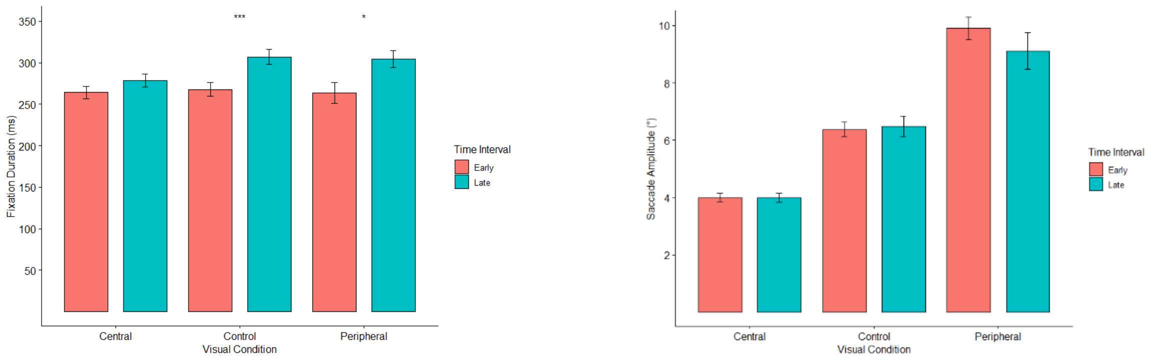

Figure 3. Mean fixation durations (A) and saccade amplitudes (B) by visual condition and time interval. Red bars indicate early time intervals (0-2 s), while blue bars indicate late time intervals (6-8 s) during scene viewing. Comparisons represent pairwise comparisons between early and late time intervals for each visual condition. Error bars indicate one standard error. * indicates p < .017. *** indicates $p < .0003$.

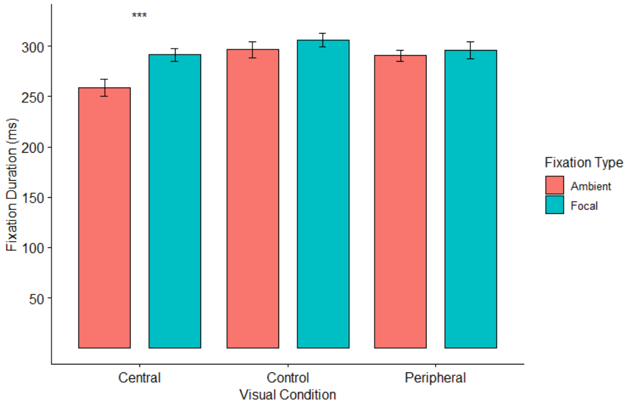

Figure 4. Mean fixation durations by visual condition and fixation type. Red bars represent ambient fixations, while blue bars indicate focal fixations. Error bars represent one standard error. *** represents $p < .0003$.
