# Supplementary material for: How central and peripheral vision influence focal and ambient processing during scene viewing
Source: J Vis. 2022 Nov 2;22(12):4. doi: 10.1167/jov.22.12.4 (PMC9639699; doi:10.1167/jov.22.12.4)
Supplement: Supplement 2 [file jovi-22-12-4_s002.pdf]

Supplementary Text

Pilot Study

Methods

With the exception of the following changes, the methodology for the pilot study was the same as the main experiment.

Stimuli and Procedure

The pilot study used a different stimulus set than the main experiment. Ninety images of natural and man-made scenes were gathered from the SUN database and from Flickr. Natural scenes consisted of bodies of water (i.e., lakes, rivers) and forests, while man-made scenes consisted of streets and housing interiors. Scenes were converted to greyscale and equalized on spatial frequency and luminance using MATLAB. Scene presentation was conducted on a CRT monitor with a refresh rate of 60 Hz. An SR Eyelink 1000 system recorded gaze monocularly at 1000 Hz.

Participants

A total of 16 undergraduates with normal or corrected-to-normal vision participated in this study.

Data Analysis

Blinks were identified as periods in which pupil information was missing. Saccades or fixations occurring within a 100 ms interval of a blink were discarded. Fixations longer than 1000 ms and shorter than 80 ms were also removed from the dataset. Fixations and saccades were only counted if they occurred in both eyes. Trials in which the total blink time was equal to or greater than 4 s (equal to 20% of the trial length) were excluded. A Greenhouse-Geisser correction was applied for repeated-measures ANOVAs where violations of sphericity were present.

Results

Visual Field, Scene Type, and Fixations/Saccades

Figure 1A depicts the change in mean fixation durations over time during scene viewing for each visual condition, while Figure 1B depicts the change in mean saccade amplitudes. A repeated-measures ANOVA tested the effects of visual condition (central, control, peripheral) and scene type (natural, urban) on fixation durations (see Figure 2A). There was a significant effect of visual condition ($F(2,30) = 3.54$, $p = .042$, $\eta^2_p = .19$) and an effect of scene type ($F(1,15) = 5.70$, $p = .031$, $\eta^2_p = .28$), such that natural scenes ($M = 299.8$ ms) involved longer fixations than urban scenes ($M = 292.6$ ms). A scene x visual condition interaction ($F(1.24,18.64) = 1.34$, $p = .28$, $\eta^2_p = .08$) was not significant. A set of Bonferroni-corrected planned comparisons were carried out ($\alpha = .017$). Fixation durations were larger in the control condition ($M = 305.9$ ms) than in the central vision condition ($M = 283.5$ ms), $t(15) = 3.49$, $p = .003$. There were no differences between the peripheral vision ($M = 298.9$ ms) and either the control ($t(15) = 0.70$, $p = .50$) or central vision conditions ($t(15) = 1.73$, $p = .10$).

Planned comparisons (Bonferroni-corrected $\alpha = .017$) found an effect of scene on fixation duration for the control condition ($t(15) = 5.65$, $p < .001$), such that natural scenes ($M = 312.5$ ms) involved longer fixations than urban scenes ($M = 299.1$ ms). There were no differences by scene for either the central vision (*Ms* = 284.7, 282.4 ms) or peripheral vision conditions (*Ms* = 302.1, 296.1 ms), both *ps* > .45.

For saccade amplitudes (see Figure 2B), there were significant effects of visual condition ($F(1.46,21.91) = 125.26$, $p < .001$, $\eta^2_p = .89$) and scene type ($F(1,15) = 11.70$, $p < .001$, $\eta^2_p = .44$), such that saccade amplitudes were larger for natural ($M = 6.8°$) than urban scenes ($M = 6.4°$). These effects were qualified by a significant scene x visual condition interaction ($F(2,30) = 12.53$, $p < .001$, $\eta^2_p = .46$). Bonferroni-corrected ($\alpha = .017$) planned comparisons indicated that saccade amplitudes were larger in the peripheral vision condition t($M = 9.2°$) than either the central vision ($M = 4.1°$) or control conditions ($M = 6.3°$). Saccade amplitudes were larger in the control condition than in the central vision condition (all *p*s < .001). Simple effects tests (Bonferroni-corrected $\alpha = .017$) found that natural scenes had larger saccade amplitudes than urban scenes for the control (*M*s = 6.8°, 5.9°), $F(1,15) = 19.20$, $p < .001$, $\eta^2_p = .56$, and central vision conditions (*M*s = 4.4°, 3.8°), $F(1,15) = 31.30$, $p < .001$, $\eta^2_p = .68$. There were no

significant effects for the peripheral vision condition (*M*s = 9.1°, 9.4°), $F(1,15) = 2.00$, $p = .18$, $\eta^2_p = .12$.

Visual Field and Early/Late Processing

A two-way repeated-measures ANOVA tested the effects of time interval (2: early, late) and visual condition (3: control, central, and peripheral) on fixation durations (see Figure 3A). Early time intervals consisted of the first two seconds of scene presentation, while late time intervals consisted of the time interval 6-8 seconds after scene presentation, as in the main experiment. There was a significant effect of time interval ($F(1,15) = 24.66$, $p < .001$, $\eta^2_p = .62$), but not visual condition ($F(2,30) = 1.38$, $p = .27$, $\eta^2_p = .08$), or the time x visual condition interaction ($F(2,30) = 2.59$, $p = .09$, $\eta^2_p = .15$). Planned comparisons ($\alpha = .017$) indicated that fixation durations were shorter during early time intervals than late time intervals for the central vision condition (*M*s = 264.1, 278.6 ms), $F(1,15) = 6.79$, $p = .020$, $\eta^2_p = .31$, but not significant post-Bonferroni correction. Effects were in a similar direction and significant for the peripheral vision (*M*s = 263.5, 304.3 ms), $F(1,15) = 8.18$, $p = .012$, $\eta^2_p = .35$, and the control conditions (*M*s = 267.6, 307.1 ms), $F(1,15) = 27.5$, $p < .001$, $\eta^2_p = .65$.

During early time intervals, post-hoc contrasts ($\alpha = .017$) did not find any significant differences for fixation durations between the central vision, peripheral vision, and control conditions (all *p*s > .68). During late time intervals, post-hoc contrasts ($\alpha = .017$) indicated that the central vision condition involved significantly shorter fixations than the control condition ($t(15) = 3.47$, $p = .003$), but did not differ significantly from the peripheral vision condition ($t(15) = 2.47$, $p = .026$). In addition, there were no significant differences in fixation lengths between the peripheral vision and control conditions for late intervals ($t(15) = 0.23$, $p = .82$).

For saccade amplitudes (see Figure 3B), a repeated-measures ANOVA found significant effects of visual condition ($F(1.37,20.58) = 144.46$, $p < .001$, $\eta^2_p = .91$), but not for time interval ($F(1,15) = 1.55$, $p = .23$, $\eta^2_p = .09$). There was a significant visual condition x time interaction ($F(2,30) = 3.81$, $p = .033$, $\eta^2_p = .20$). Simple effects tests ($\alpha = .017$) found that effects of time interval for the peripheral vision condition were not significant ($F(1,15) = 4.02$, $p = .063$, $\eta^2_p = .21$), even though the magnitude of saccade amplitudes was larger during early time intervals (*M*

= 9.9°) than late time intervals (*M* = 9.1°). Effects were not significant for central vision (*M*s = 4.0°, 4.0°), $F(1,15) = 0.00$, $p = .98$, $\eta^2_p = .00$, or for control (*M*s = 6.4°, 6.5°), $F(1,15) = 0.23$, $p = .64$, $\eta^2_p = .02$. Planned comparisons ($\alpha = .017$) tested whether visual condition affected saccade amplitudes during early and late time intervals. During early time intervals, there were significantly larger saccades for peripheral vision when compared to control or central vision, while the control condition also involved larger saccades than central vision (all *p*s < .001). During late time intervals, an identical pattern of significant differences (saccade amplitudes: peripheral > control > central) was found (all *p*s < .001).

Focal and Ambient Fixations

Fixations were classified as ambient or focal on the basis of their preceding saccade, as per Pannasch & Velichkovsky (2009). Ambient fixations were preceded by a saccade with an amplitude > 5°, and focal fixations were preceded by a saccade with an amplitude <5°. A two-way repeated-measures ANOVA indicated significant effects of visual condition ($F(2,30) = 4.71$, $p = .017$, $\eta^2_p = .24$) and fixation type ($F(1,15) = 13.21$, $p = .002$, $\eta^2_p = .47$) on fixation durations, with ambient (*M* = 286.0 ms) fixations being shorter compared to focal (*M* = 301.8 ms) fixations (see Figure 4). There was also a significant visual condition x fixation type interaction ($F(2,30) = 10.74$, $p < .001$, $\eta^2_p = .42$). Simple effects tests ($\alpha = .017$) found a significant effect on fixation type on fixation duration for central vision ($F(1,15) = 37.0$, $p < .001$, $\eta^2_p = .71$), such that ambient fixations were shorter (*M* = 260.8 ms) than focal fixations (*M* = 293.4 ms). There were no significant effects for the control condition ($F(1,15) = 3.41$, $p = .085$, $\eta^2_p = .19$) or peripheral vision ($F(1,15) = 1.04$, $p = .32$, $\eta^2_p = .07$). Durations were shorter for ambient fixations compared to focal fixations for both the control condition (*M*s = 300.5, 308.3 ms) and peripheral vision (*M*s = 296.5, 303.7 ms), but differences were not significant.
